# Response to: Does COVID-19 pose a challenge to the diagnoses of anxiety and depression? A psychologist's view

**DOI:** 10.1192/bjb.2021.118

**Published:** 2022-02

**Authors:** Allan House

**Affiliations:** Emeritus Professor of Liaison Psychiatry, University of Leeds, Leeds, UK. Email: a.o.house@leeds.ac.uk

In her recent article, Johnstone (Bulletin, September 2021) writes critically about how we have responded professionally to the effects of the COVID-19 pandemic. While it is easy to agree with some of what she has to say, much of her argument consists of a series of assertions that are neither entirely accurate nor logically connected to each other or to her main contention.

## What is the problem?

Johnstone's central claim is that by using psychiatric diagnosis we label things as abnormal that are in fact normal. The opening example of handwashing and cleaning is unfortunate because it is unconvincing – hardly anybody spends ‘most of the day’ doing it and there is more to the diagnosis of OCD than cleaning: resistance, ritualising, other compulsions and so on. Certainly, the use of florid metaphors about tsunamis and pandemics of mental disorder is unhelpful, and research does show that much unhappiness and anxiety during the pandemic has proved to be transient. But then many illnesses, including those caused by the COVID-19 virus, can be transient and non-disabling – it doesn't mean they aren't illnesses. Every doctor, including every psychiatrist, knows the value of watchful waiting: the question is how we respond when symptoms are not transient or non-disabling. When Johnstone talks about ‘people with a psychiatric history’, I take it to mean people who most psychiatrists would regard as having a long-term mental disorder. We can agree that ‘It is untrue and even patronising to assume that everyone in this group will fail to cope’, but does any psychiatrist actually assume that? More fundamentally, Johnstone is opposed to the idea of psychiatric diagnosis (and not just of anxiety and depression) because it rests upon defining mental illness in relation to social norms while (as she and her colleagues have argued elsewhere) masquerading as being analogous to the more legitimate processes of medical diagnosis. It is an error to assume that medical diagnosis is radically different in all respects: it does for example recognise social causes (cigarette smoking, hazardous drinking, unhealthy eating, physical inactivity) and defines some of its most prevalent disorders such as hypertension, hyperlipidaemia and diabetes mellitus according to deviation from norms. More important is the question of whether the states so diagnosed are harmful and, if so, whether intervening is beneficial.

## Who is responsible?

I found it difficult to suppress a smile at Johnstone's jibe about the self-serving nature of articles promoting the importance of research in the areas of expertise of the authors. But it is too narrow to name only academics and Public Health England as the actors in a debate about the nature of public mental health – professional bodies, the pharmaceutical industry, politicians and journalists are among others who set the agenda and the tone. The emergence of the phrase ‘mental health’ is an interesting topic in its own right, and one way to view its effects is to see it as a vehicle for medicalisation of distress. It might, however, be as useful to think of it as effect rather than cause of the individualisation of societal problems – a phenomenon that has deep cultural roots and consequences that go beyond psychiatry into penal policy, welfare provision and education.

## What are the alternatives?

It is a category error to propose formulation as an alternative to diagnosis – the latter is a descriptive statement, whereas the former provides an explanatory framework, a point illustrated by the training requirement that psychiatrists are expected to be able to make a biopsychosocial formulation and management plan as well as coming to a diagnosis. It is not news that the onset of many mental disorders is preceded by adverse life events and difficulties – research in this area goes back half a century – or that the content of some people's illnesses reflects these experiences. However, bundling together all mental disorders as ‘various forms of distress…that are understandable responses to adversities’ does not do justice to the issues. Not everybody reports life adversities before onset; the nature of adversity may be reflected in the content of some but not all conditions; life adversity does not explain the differences in form of the various mental disorders; there is a strong genetic risk for some disorders. It is difficult to know what it means to say that mental disorder is ‘what we do’ in response to threats, but in my reading it is hard to see it as other than dismissive of the reality of mental illness.

## Collective trauma and a collective response

There is a disconcerting *volte face* at the end of Johnstone's piece. Having presented the argument that what we are seeing in the pandemic is essentially normal, part of a meaningful response to stress and not to be pathologised, we are finally offered the idea of collective trauma – defined as an experience that overwhelms our usual ways of coping. If states like anxiety and depression are to be thought of as arising because of this overwhelming of usual ways of coping, how is that different from the way that psychiatrists think about what they are likely to call mental disorders? Only, it seems, in the reluctance to use a descriptive vocabulary that distinguishes between different conditions – as if it is a trivial matter whether somebody is hearing voices, embarked upon life-threatening self-starvation or unable to touch a newspaper for fear it will give them a fatal infection. How are we supposed to use this way of thinking to help people now, while they and we wait for a fairer society? Local peer networks may indeed help some, but they won't suffice for the severity and diversity of problems we face. One of the central tensions of healthcare is that we can recognise that health and illness have social determinants, but as clinicians it is individuals that we see. It isn't a question of picking one or the other – both are important, and I think most psychiatrists understand that.

## A conclusion

Surely we can all agree about some things: it is important not to medicalise distress that does not merit such an approach; social adversities are important risks to our mental well-being, and government policies in recent years have both exacerbated these risks and done much damage to society's ability to help those most in need as a result of them; professionals in healthcare have a responsibility to speak out both for individuals in need and also about the social conditions that contribute to their difficulties. These simple and powerful messages are obscured by wrapping them, as here, in a muddled polemic animated as much as anything else by anti-psychiatry sentiment.

